# Revisiting the Latency of Uridine Diphosphate-Glucuronosyltransferases (UGTs)—How Does the Endoplasmic Reticulum Membrane Influence Their Function?

**DOI:** 10.3390/pharmaceutics9030032

**Published:** 2017-08-30

**Authors:** Yuejian Liu, Michael W. H. Coughtrie

**Affiliations:** Faculty of Pharmaceutical Sciences, The University of British Columbia, Vancouver, BC V6T 1Z3, Canada; yuejian.liu@alumni.ubc.ca

**Keywords:** UDP-glucuronosyltransferase, latency, microsomes, glucuronidation, regulation

## Abstract

Uridine diphosphate-glucuronosyltransferases (UGTs) are phase 2 conjugation enzymes mainly located in the endoplasmic reticulum (ER) of the liver and many other tissues, and can be recovered in artificial ER membrane preparations (microsomes). They catalyze glucuronidation reactions in various aglycone substrates, contributing significantly to the body’s chemical defense mechanism. There has been controversy over the last 50 years in the UGT field with respect to the explanation for the phenomenon of latency: full UGT activity revealed by chemical or physical disruption of the microsomal membrane. Because latency can lead to inaccurate measurements of UGT activity in vitro, and subsequent underprediction of drug clearance in vivo, it is important to understand the mechanisms behind this phenomenon. Three major hypotheses have been advanced to explain UGT latency: compartmentation, conformation, and adenine nucleotide inhibition. In this review, we discuss the evidence behind each hypothesis in depth, and suggest some additional studies that may reveal more information on this intriguing phenomenon.

## 1. Introduction

**Figure 1:**
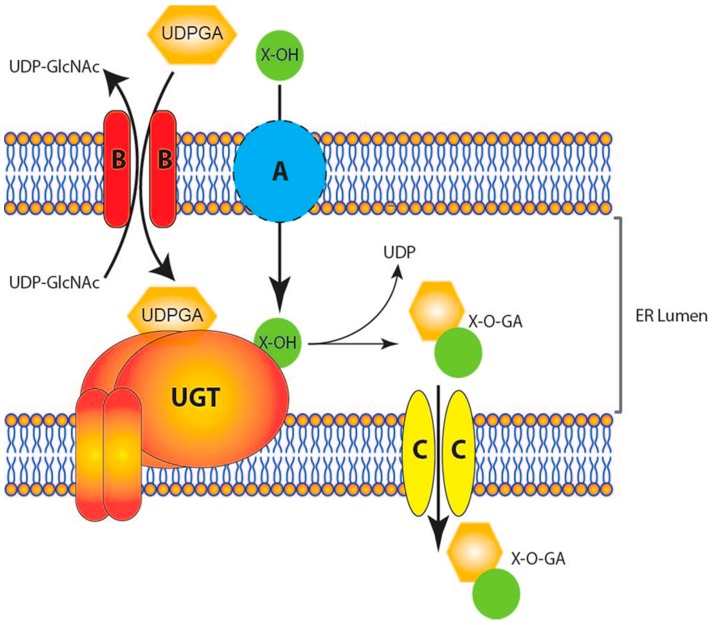
The glucuronidation system in the endoplasmic reticulum. Substrates (X-OH) enter the lumen by diffusion (A), and UDPGA is transported via the UDPGA/UDP-GlcNAc antiporter (UGTrel7, B). Following the conjugation reaction, the glucuronide products (X-O-GA) are removed from the lumen by glucuronide transporter(s) (C). UDPGA: Uridine diphosphate-glucuronic acid; UDP-GLcNAc: UDP-*N*-acetylglucosamine.

Uridine diphosphate-glucuronosyltransferases (UGTs) comprise a superfamily of phase 2 conjugation enzymes that catalyze the glucuronidation of numerous substrates at functional groups such as –OH, –COOH, –NH_2_, –SH, and C–C [1]. They are arguably the most important conjugation enzymes facilitating the excretion of various endobiotics such as bilirubin, steroid and thyroid hormones, bile acids, and retinoids, as well as xenobiotics including environmental chemicals, pollutants, and drugs [2]. These substrates are mostly hydrophobic and are conjugated by the UGTs with the glucuronic acid moiety derived from the co-substrate, uridine diphosphate-glucuronic acid (UDPGA). The resulting glucuronide conjugates that are generally more polar and water-soluble are substrates for numerous membrane transporters and thus they can eventually be excreted out of the body in bile and urine (Figure 1).

UGTs are type-I transmembrane glycoproteins mainly located within the smooth endoplasmic reticulum (ER), although some isoforms can be found in the nuclear envelope [3,4]. UGTs demonstrate protein–protein interactions within the superfamily (homo- and hetero-dimers and possibly higher order structures) or with other enzymes such as the cytochromes P450 [5,6,7,8,9,10,11,12], as demonstrated by studies using co-immunoprecipitation, fluorescence microscopy, and Förster resonance energy transfer (FRET), among many other approaches (as summarized in [6]). The interactions have been shown to alter kinetic properties of UGTs [7,9,10].

Detoxification is divided into two phases [13]. The basic principle is that oxidative metabolism by phase 1 enzymes (such as cytochromes P450, CYP) results in the addition or revealing of a functional group (e.g., –OH, –COOH or –NH_2_) such that the compounds can be conjugated by phase 2 enzymes such as the UGTs. This necessitates the phase 1 metabolites crossing the ER membrane in order to access the UGT active site. Evidence suggests a functional interaction between the UGT and CYP proteins to facilitate the multistep detoxification process [5,12,14,15].

Besides detoxification, glucuronidation can activate substrates such as certain procarcinogens, as well as morphine that can be converted to morphine 3- and 6-glucuronides, where the latter is a more potent analgesic than the parent drug [16]. Glucuronidation also produces toxic metabolites such as steroid D-ring glucuronides that may be responsible for intrahepatic cholestasis of pregnancy [17]. The human UGT superfamily is divided into five subfamilies: UGT1A, 2A, 2B, 3A, and 8. They are mainly expressed in liver, but are also prevalent in extrahepatic tissues including gastrointestinal tract, kidney, lung, brain, and reproductive tissues [1,18]. The human UGT1A isoforms are generated from a single gene located at chromosome 2q37.1 through alternative splicing. Specifically, there are 13 first exons, including 4 pseudogenes, each of which is controlled by an individual promoter (1A1, 1A2p, 1A3, 1A4, 1A5, 1A6, 1A7, 1A9, 1A13p, 1A10, 1A8, 1A11p, and 1A12p). These 13 exon/promoter pairs are upstream of a second region that consists of 5 common exons under the regulation of a single promoter (exons 2, 3, 4, 5a, and 5b). The mRNA for each UGT1A isoform is generated by splicing one of the first exons with the group of common exons. This mechanism of alternative splicing is conserved in human, mouse, and rat UGTs [19].

## 2. UGTs and Latency

The majority of the UGT enzyme protein, including its catalytic site, is believed to reside inside the ER lumen (and partially associate with the ER membrane); thus there are physical and physiological barriers to substrates accessing the active site of the enzymes within the ER lumen, as well as to the egress of reaction products from the ER and, subsequently, from the cell. This leads to the phenomenon of latency of UGT activity in preparations of ER (i.e., microsomes). The phenomenon of latency derives from experimental observations that in isolated microsomal preparations, UGTs generally show increased activities after the membranes are physically or chemically disrupted [20,21,22,23,24,25,26,27,28]. Other enzymes present in the lumen of the ER, such as glucose-6-phosphatase, also display latency [29]. Although the latency of UGTs has been known for more than half a century, the exact mechanism(s) underlying it remain incompletely understood, and this has generated several hypotheses and much heated debate during this time. The phenomenon of latency is an important factor in the consistent underprediction of drug clearance in vivo based on in vitro measurement of UGT activity [30], since it is of particular importance to be able to accurately measure UGT activity in microsomal preparations. Examples of treatments that disrupt microsomal membranes and reveal UGT latency (to different extents) include grinding in sand, sonication, high hydrostatic pressure, detergents, phospholipase A or C, staphylococcal *α*-toxin, or the pore-forming antibiotic alamethicin [22,23,28,31,32,33,34,35,36,37,38,39]. It has been postulated that these agents, or physical stressors, act in different ways, for example by allowing unlimited access of substrate and co-substrate to the enzyme’s catalytic site; by inducing an alteration in the conformation of the enzyme; by releasing UGT inhibitors from the microsomal lumen; or a combination of these mechanisms which of course are all dependent to some extent on the ER membrane.

Three major hypotheses have been developed over the years to explain latency: compartmentation, conformation, and adenine-nucleotide inhibition. The first two hypotheses mainly address whether the ER membrane limits UGT activity by acting as a barrier blocking access of UDPGA (and potentially aglycone substrate) to the luminal space and thus the UGT active site, or by regulating the conformation of the UGT protein through lipid–protein (or potentially protein–protein) interactions. The last hypothesis was the most recently proposed, based on the inhibitory effects of adenine-containing nucleotides on the UGTs. Under this hypothesis, the membranes might limit UGT activity by sequestering these natural inhibitors in the lumen.

The latency phenomenon makes accurate measurement of UGT activity in microsomal preparations challenging, however it is one of the most important factors to take into consideration in the design of UGT enzyme assays. Different methods of preparation of the microsomal fraction can have major impact on measurable latency, since the level of intactness of the membrane preparation clearly influences the enzyme activity. For example, it has been shown that preparation of microsomal fractions from freshly isolated liver is significantly preferable over frozen tissue, which tends to produce microsomal fractions with much lower levels of intactness [40]. Similarly, microsomes prepared from rat lung tissue exhibited far lower latency levels than those from rat liver, although this may derive from the harsher treatment required to homogenize rat lung tissue [41]. The nature of the disrupting agent also has an impact on the latency—for example some detergents appear to be much harsher than others, resulting in inhibition of UGT activity. In our experience, Lubrol PX has been the most effective detergent for the disruption of the microsomal membrane, and for the solubilization of the UGT protein from the microsomal membrane for purification (e.g., [41,42]). It is generally accepted that the pore-forming antibiotic alamethicin is the membrane disruptor of choice for UGT assays since the UGT enzymes seem less sensitive to variations in its concentration than they are to various detergents [34]. Where liver microsomal preparations are used to generate glucuronide metabolites at a semi-preparative scale, for example for metabolite identification, the issue of latency is also important. For instance, long incubations in the presence of detergents could result in inhibition of the UGT activity, so careful choice of membrane perturbation method would be of particular importance.

Here we review the major hypotheses that have been proposed to explain the phenomenon of latency as it applies to UGTs, and discuss future studies that might be needed to gain a greater understanding of its origins and implications.

## 3. Compartmentation Hypothesis

This hypothesis is based on the assumption that the highly hydrophilic co-substrate for the glucuronidation reaction, UDPGA, (and possibly certain aglycone substrates) is not able to diffuse across the ER membrane, and therefore that disruption of the membrane integrity is required to reveal the full extent of in vitro UGT activity in microsomal preparations. The discovery of transporters for nucleotide sugars, including UDPGA, has provided very strong evidence in support of this hypothesis.

The barrier function of microsomal membranes was proposed by Winsnes in 1972 [38] and was later elaborated by Berry and Hallinan, who firstly proposed the compartmentation model to explain latency based on previous experimental results [43]. In this model, UGT located on the luminal side of the ER is partially embedded in the membrane, together with a transmembrane UDPGA permease and a lumenal nucleoside diphosphatase (NDPase). This model predicts that UDPGA needs to be transported by the permease into the microsomal lumen where UGT catalyzes glucuronidation reactions, then the NDPase hydrolyzes UDP, a co-product besides the glucuronide conjugate, to facilitate the forward reaction since UDP is an inhibitor of UGTs. Later evidence strongly supports this model, including the prediction of UGT topology and the discovery of UDPGA transporters.

The identification of complete cDNA sequences improved the understanding of many aspects of UGTs such as the ER targeting process and topology [1]. Initial reports on the sequences came from Jackson et al. and Mackenzie et al. for rat liver UGTs [44,45], and allowed researchers to synthesize UGT proteins for studies on post-translational modifications [1,46,47]. In general, UGTs are synthesized as precursors of approximately 530 amino acid residues, and are targeted to the ER by an N-terminal signal sequence. A C-terminal di-lysine motif has been found in several human UGT sequences, and could act as an ER retention signal [48]. Following membrane integration, the N-terminal signal peptide is cleaved and the enzyme is *N*-glycosylated in the ER lumen. Notably, other internal topological elements within the UGTs might be required to regulate the integration process, as demonstrated for UGT1A6 [49,50]. In this way, the UGTs become mature ER enzymes of just over 500 residues [1]. Although significant portions of the protein may associate with the membrane, approximately 95% of the polypeptide chain is predicted to be luminal, and is connected to the cytosolic C-terminal tail through a 17-residue alpha helix that spans the lipid bilayer.

Although the intimate association between UGT protein and the ER membrane has so far prevented the generation of a complete crystal structure, the predicted topology of UGTs has been well developed. Vanstapel and Blanckaert predicted the catalytic site of bilirubin UGT faces ER lumen [51], which was followed by the development of a more complete topology model by Shepherd et al. [52]. This model was proposed based on the evidence from the authors’ own results indicating that the majority of bilirubin UGT (UGT1A1) was not exposed to the cytosol, hydropathy plot analysis [53], and complete cDNA sequences of androsterone UGT [54,55], and rat phenol UGT (UGT1A6) [56]. Site-directed mutagenesis experiments have also confirmed that the UGT proteins are comprised of two domains, one binding the UDPGA (in the conserved C-terminal part of the protein) and one that presumably confers substrate preference located in the less-well conserved N-terminal region [57]. The determination of an X-ray crystal structure (1.8-Å resolution) for the C-terminal domain of human UGT2B7 is another strong piece of evidence confirming the presence of a nucleotide-binding site in the C-terminal half of the UGT proteins [58]. Above all, the sequence information together with recombinant DNA technology have allowed the development of a topology model that is generally accepted today (Figure 1). Compared to the topology proposed by Shepherd et al. [52], the most significant advances include the more specific locations of substrate and co-substrate binding sites, and the presence of transporters for UDPGA and glucuronides. If the binding sites are luminal as predicted, then the rate of glucuronidation might be constrained by the membrane barrier limiting the access of UDPGA, a highly charged molecule synthesized in the cytoplasmic space [59], to the reaction center. Although the complete crystal structure of a UGT protein is unsolved, homology modeling has been used to predict the three-dimensional structure [57]. For example, human UGT1A1 and UGT1A10 were homology-modeled using plant UGT71G1 and UDP-galactose 4-epimerase from *Escherichia coli*, respectively [60,61].

In addition, a transporter (or transporters) might be needed to facilitate the efflux of polar glucuronides out of the ER; this transporting activity has been biochemically demonstrated with a rate comparable to the rate of glucuronide synthesis [62]. Interestingly, the authors suggested that glucuronide efflux could be another rate-limiting step besides UDPGA import, because the rate of glucuronidation is highly variable among intact microsomes and it is unlikely that transport of UDPGA, the universal co-substrate for all glucuronidation reactions, is responsible for all of the variation observed. The identity of glucuronide efflux transporter(s) is still unknown and in contrast to this, UDPGA transporters have been intensively studied and identified, as described below. The role of UDP-*N*-acetylglucosamine (UDP-GlcNAc) has been key to advancing the compartmentation hypothesis.

It is well known that UDP-GlcNAc can activate UGT activity in microsomal preparations in the presence of UDPGA, having been first demonstrated by Pogell and Leloir, who suggested the effect was partly due to inhibition of the breakdown of UDPGA [24]. Later work suggested that UDP-GlcNAc acted as an allosteric effector of UGT [63]. However, it was Berry and Hallinan who proposed the existence of a “UDPGA permease” [43], following on from the suggestion made by Winsnes that UDP-GlcNAc might increase the permeability of the microsomal membrane to UDPGA, thus activating the enzyme [38]. UDP-GlcNAc stimulated UGT activities only when UDPGA was added to preparations of intact microsomes, but not when it was generated in situ by trans-glucuronidation or reverse glucuronidation reactions [64,65], which indicates the stimulation effect of UDP-GlcNAc occurs at the step of UDPGA transport into microsomes. The integrity of microsomal membranes needs to be maintained for UDP-GlcNAc stimulation of UGT activity, because UDP-GlcNAc no longer activated UGTs when microsomal membranes were disrupted by Triton X-100 treatment, sonication, or Lubrol PX treatment [28,31,41]. Interestingly, UGT activity in rat lung microsomes (with 1-naphthol as substrate) was insensitive to UDP-GlcNAc stimulation, unlike in rat liver microsomes [41], although this may be due to excessive mechanical disruption of the membranes during homogenization resulting from the nature of the lung tissue. Taken together, this evidence suggested that UDP-GlcNAc activates UGTs through an indirect mechanism that requires an intact microsomal membrane, presumably by enhancing the transport and access of UDPGA to the catalytic site. Evidence supporting this presumption includes the observation that glucuronidation rate was increased by UDP-GlcNAc when the concentration of UDPGA was high or saturating [24]. Additionally, Berry and Hallinan demonstrated that UDP, UTP, and UDP-glucose showed inhibitory effects on UGT activities only after microsomal membranes were disrupted [43], whereas native UGTs were resistant to the inhibitors, suggesting that these compounds are not transported by the presumptive UDPGA permease [63,66]. Therefore, it was concluded that the presumptive permease has a regulatory role in UGT activities by selectively transporting UDPGA.

Further evidence came from the observations that addition of *N*-ethylmaleimide (NEM) blocked the activation effect of UDP-GlcNAc on native UGT activity within intact microsomes, without a direct inhibition on the enzyme’s catalytic site [67]. This piece of evidence suggested the presence of membrane permease-containing thiol groups that are required for the action of UDP-GlcNAc. Indeed, later studies showed that NEM inhibited UDPGA transport into the ER of permeabilized rat hepatocytes, which in turn inhibited glucuronidation of 4-methylumbelliferone (4-MU), a non-selective substrate metabolized by many UGT isoforms [68,69]. However, when microsomal and ER membranes were permeabilized, glucuronidation was not affected by NEM treatment [69].

The discovery of UDPGA transporters was preceded by studies recognizing a carrier-mediated, UDPGA transport activity at the rat liver ER membrane [68,69,70,71]. The common question raised by these studies was as to how important UDPGA transport could be to glucuronidation reactions. Bossuyt and Blanckaert carried out a series of studies characterizing the transport activities of UDPGA and UDP-GlcNAc across microsomal membranes [68,72], and were able to connect the two transport systems when UDPGA uptake into the microsomes preloaded with UDP-GlcNAc showed an overshoot effect [69]. Hence, UDP-GlcNAc might be exported by a bidirectional carrier to trans-stimulate the import of UDPGA for UGT catalysis. It turned out that UDPGA transport was both required and necessary for glucuronidation, because the inhibition or stimulation of UDPGA transport by NEM or UDP-GlcNAc, as partly mentioned above, inhibited or stimulated glucuronidation respectively towards 4-MU in microsomes and permeabilized hepatocytes [73]. Indeed, UDPGA transport seemed to be the rate-limiting step of glucuronidation reactions, which formed the basis of the compartmentation hypothesis in that the release of the transport constraint would significantly increase the glucuronidation rate.

A number of nucleotide sugar transporters (NSTs) have now been cloned, expressed and functionally characterized. UDP-galactose transporter-related protein 7 (UGTrel7), a human NST, was shown to transport both UDPGA and UDP-*N*-acetylgalactosamine (UDP-GalNAc) into microsomal vesicles when expressed in *Saccharomyces cerevisiae* [74]. Kobayashi et al. molecularly and functionally characterized four human NSTs, namely UGTrel1, UGTrel7, huYEA4, and huYEA4S, the last two being newly identified human NSTs that are splice variants of each other [75]. These transporters all mediated UDP-GlcNAc-dependent UDPGA uptake into the microsomes isolated from recombinant V79 cells expressing the NST cDNAs, although UGTrel7 was by far the most efficient. These authors also demonstrated that UGTs themselves, at least UGT1 family members, were not significantly involved in the transport of UDPGA. Muraoka et al. subsequently confirmed that UGTrel7 (expressed in *S. cerevisiae*) transported UDPGA in a manner that was much enhanced by UDP-GlcNAc and showed that UGTrel7 was a UDPGA/UDP-GlcNAc antiporter [76]. Therefore, after more than 50 years since the discovery that UDPGA was the co-substrate and glucuronic acid donor for UGTs [77,78], these two papers provided the most direct evidence of how the highly charged co-substrate is delivered to the UGTs in the microsomal lumen, and how UDP-GlcNAc enhances this process. The rapid advance in gene editing technology, such as the CRISPR/Cas9 system, provides an alternate approach to investigating the role of UDPGA transporters in the latency of UGTs. It should be possible, for example, to delete a UDPGA transporter such as UGTrel7 in a cell line (or embryonically in an animal model) to determine the contribution of individual NSTs to UGT latency.

UDPGA transport is a complex process, and probably involves other transport molecules, as described by Rowland et al. who demonstrated that UDPGA uptake into human liver microsomes involves more than one transporter [79]. Other NSTs capable of transporting UDPGA at ER membrane have been identified. For instance, Selva et al. and Goto et al. identified the gene fringe connection (*frc*) that encodes an NST transporting UDPGA, UDP-GlcNAc, and UDP-xylose from the cytoplasm to the lumen of ER/Golgi in *Drosophila* [80,81]. Interestingly, this transporter is important in growth-factor signaling pathways including Wnt/Wingless-, Hedgehog-, fibroblast growth factor-, and Notch-dependent pathways, by transporting UDP-sugars into the Golgi to facilitate the synthesis of heparan sulfate proteoglycan and the glycosylation and maturation of Notch receptor. Lastly, Suda et al. identified the gene *hfrc 1* homologous to *frc* (*Drosophila melanogaster*), *sqv-7* (*Caenorhabditis elegans*), and *UGTrel7* (human) [82]. It encodes an NST that is located at the Golgi and transports UDP-GlcNAc, UDP-glucose, and UDP-mannose.

## 4. Conformation Hypothesis

The concept that the conformation of the UGT enzyme protein is subject to influence by the hydrophobic lipid environment in the ER membrane, and it is this that controls the activity of enzymes, is the underlying basis of the conformation hypothesis. In contrast to the compartmentation hypothesis, the conformation hypothesis explains latency by assuming that the catalytic site of UGTs is accessible by substrates and UDPGA and the membrane disruption induces the formation of different kinetic forms of the enzyme with increased activity [39]. 

According to Vessey and Zakim, treatments altering the lipid portion of bovine liver microsomes increased UGT activity at *V*_max_ towards *p*-nitrophenol, including the addition of phospholipase A or Triton X-100, and sonication [37]. These treatments can alter the lipid composition as well as the integrity of the microsomal membrane. For instance, phospholipase A produces lysophospholipids that can act as detergents when present at high concentrations within the membrane. Therefore, the increase in *V*_max_ of UGTs could be due to a change in the protein–lipid interaction or greater access of substrates to the catalytic site after the membrane integrity had been lost, or a combination of both. Because Triton treatment only sped up the forward reaction which is the glucuronidation reaction catalyzed by UGTs [37], the activation effect was restricted to the UGTs (not other microsomal components) when the luminal space was fully accessible. Besides, phospholipase A treatment has been shown to alter the binding specificity for UDPGA, allowing for the competitive binding from other UDP-sugars to the UGTs [63,83]. Hence, phospholipase A seems to induce a change in the conformation of the co-substrate binding site that can fit the structures of other UDP-sugars. Overall, these experiments suggested that the effect of membrane perturbants is mainly the alteration of lipid composition which directly affects the enzyme itself, as Zakim and Dannenberg argued [39]. They concluded that the enzyme might exist in different functional states demonstrating different kinetic properties, and the lipid environment controls the enzyme activity by intimately regulating its conformation.

The interaction between substrate and co-substrate binding sites within a single UGT protein was another piece of evidence used to support the existence of multiple conformational forms [84,85]. Vessey and Zakim proposed that *p*-nitrophenol UGTs follow a random order kinetic mechanism after they carried out product inhibition and isotope exchange studies, and so the enzymes can firstly bind substrate or co-substrate and form rapid equilibrium [85]. Interestingly, under high concentrations of *p*-nitrophenol, UGTs showed increased values of *V*_max_ and *K*_m UDPGA_, which suggested that occupation of the substrate binding site decreased the binding affinity for the co-substrate. The interaction between substrate and co-substrate binding sites was further investigated by Hochman and Zakim who used an enzyme reconstitution system where a delipidated form of purified UGT, called GT_2p_, was reconstituted into different species of phospholipids to study their influence on the enzyme properties [84]. GT_2p_ represented the second peak of eluted UGT after a hydroxylapatite column purification from pig liver microsomes, and showed a single band of protein following sodium dodecyl sulfate—polyacrylamide (7.5%) gel electrophoresis (SDS-PAGE) [86]. It was shown that the nature of phospholipids used to reconstitute GT_2p_ activity affected the extent to which substrate binding decreased the binding affinity of the co-substrate. In parallel with this result, the α-glucuronidase activity of GT_2p_ was differentially affected by the binding of structural variants of aromatic ethers that only occupy the substrate binding site without being conjugated. Therefore, the binding of a substrate seems to induce a conformational change in the enzyme that possesses an altered co-substrate binding affinity, which is dependent on the surrounding lipid environment.

Further experiments suggested that the physical properties, rather than the chemical structures, of phospholipids surrounding UGTs seemed to play a role in the interaction with the enzymes and regulation of their kinetic patterns. For example, when GT_2p_ was reconstituted into bilayers of 1,2-dimyristoylphosphatidylcholine or 1,2-dipalmitoylphosphatidylcholine lipids in the gel phase, the enzyme displayed non-Michaelis–Menten kinetics, however when the lipids were heated to liquid–crystal phase, the enzyme(s) displayed Michaelis–Menten kinetics [87,88]. The change in enzyme kinetics was solely due to the phase transition of phospholipids independent from a direct effect of increasing temperature. Furthermore, the GT_2p_ demonstrating non-Michaelis–Menten kinetics had two different binding sites for UDPGA per molecule of enzyme, and the appearance of Michaelis–Menten kinetics after phase transition occurred was in parallel with the disappearance of one binding site [87]. These results could be explained by the conformational hypothesis in that the physical state of the phospholipids seems to determine the number of functional binding sites for UDPGA by changing enzyme conformation, although it is possible that the change in membrane fluidity promotes the association/disassembly of UGT monomers by modulating protein–protein interactions. The length and degree of saturation of phospholipid acyl chains are important for regulating membrane fluidity, and have been shown to affect the activity of UGTs [89]. For instance, for the purified pig liver GT_1p_, the first eluted fraction from a hydroxylapatite column as described by Hochman and Zakim [90], the lysophosphatidylcholine species that had longer acyl chains with more unsaturated bonds led to greater activation at *V*_max_. This result further suggested that the interaction between UGTs and phospholipids does not depend on the specific chemical structures of phospholipids. Rather, as Rotenberg and Zakim suggested, the interaction occurs through a non-specific mechanism depending on the physical state of phospholipids [91].

The experiments described above repeatedly emphasize the role of the lipid environment in the regulation of UGT conformation. The change in conformation can in turn alter the rate of glucuronidation presumably by changing the catalytic mechanism, as demonstrated for GT_2p_ reconstituted into different species of lysophosphatidylcholine [86,92]. Specifically, changing the lipids used to reconstitute GT_2p_ from oleoyl lysophosphatidylcholine to stearoyl lysophosphatidylcholine led to changes in the rate of glucuronidation depending on the specific aglycone substrates: a 20-fold decline for *p*-nitrophenol, no change for 1-naphthol and phenol, and a 3-fold decline for *p*-bromophenol. Hence, the specific lipid environment has a selective effect on the glucuronidation rate towards different aglycones. Two mechanisms have been proposed as to how the lipid–protein interaction affects the catalytic rate of UGT (in this case, purified GT_2p_). Firstly, phospholipids may regulate enzyme–substrate interactions and determine how much inherent binding energy to UDPGA can be shared for catalysis to enhance the reaction rate [92]. Only certain species of lysophosphatidylcholine could influence the enzyme in a way that more inherent binding energy is used for catalysis. Alternatively, phospholipids like oleoyl lysophosphatidylcholine speed up glucuronidation by facilitating the bond breaking between the C1 of glucuronic acid and UDP [86].

Besides kinetic properties and avidity for substrate binding, the lipid environment also regulates the thermal stability of UGTs [91]. Rotenberg and Zakim’s data suggest that the GT_2p_ fraction of UGTs existed in three forms through conformational changes: the active form (E), inactive form (E’), and denatured form. The change between E and E’ is controlled by temperature and is reversible, whereas the denatured form is obtained by heating E’ to a certain extent. The specific temperatures required for the occurrence of these conformational changes are dependent on a specific lipid environment. In general, the reconstitution of pure, delipidated GT_2p_ into different species of phosphatidylcholine, such as distearoylphosphatidylcholine, dioleoylphosphatidylcholine, or 1-stearoyl-2-oleoylphosphatidylcholine, stabilized the E form with a greater change in the entropy of the whole system rather than the enthalpy, and slowed down denaturation rate of the E’ form. However, each of these phospholipids affected the thermal stability of GT_2p_ to a different extent depending on the nature of the acyl chains, so the increase in temperature might alter enzyme conformation through modulating the physical state of phospholipids. Using the reconstitution method, Hochman and Zakim showed that only gel-phase phospholipids could reconstitute the sensitivity of the purified GT_2p_ fraction to stimulation by UDP-GlcNAc, a physiological activator of UGTs [90]. Also, GT_2p_ seems to have a favorable interaction with viscous lipids [87]. These data suggest UGTs are restricted by certain forces within a subregion of the ER membrane where the lipids are gel-like, hence it is less likely that the enzymes randomly interact with the bulk phase of lipids within the ER membrane [87,90]. The forces could come from the interactions between UGTs and specific acyl chains in the lipid bilayer, or between different UGT isoforms that preferentially aggregate in the gel-like regions.

Alternatively, the gel-like regions might be formed by lipid rafts, membrane microdomains that are enriched in cholesterol, sphingolipids, and specific proteins such as glycosylphosphatidylinositol (GPI)-anchored proteins and Src-family kinases [93]. These microdomains could be formed by the phase segregation of membrane lipids [93], and/or by anchoring of the lipids to actin filaments [94]. More importantly, the rafts have various functions including endo-, exo-, and transcytosis, polarized intracellular trafficking of specific membrane proteins, and signal transduction [93,95,96]. They can transiently recruit membrane and intracellular proteins to initiate a cellular signaling event [97]. Since the intracellular transport of UGTs to the ER membrane is not fully understood, it is likely that UGTs functionally interact with lipid rafts for the purposes of intracellular transport, ER localization, and/or regulation of the enzyme stability and activity. In vitro and in vivo experiments have demonstrated the effect of cholesterol, a major component of lipid rafts, on the activity of UGTs [98,99]. Specifically, the addition of cholesterol to guinea pig liver microsomes increased UGT activity at *V*_max_; diet supplementation that increased the membrane content of cholesterol in guinea pig liver ER showed similar results to that of the in vitro data. In both cases, the activity of UGTs was studied under the native membrane environment. Rotenberg and Zakim reconstituted delipidated GT_2p_ into different phospholipids to study the influence of the lipid environment on the observed cholesterol effect [100]. They showed that the modulation of UGT activity and stability by cholesterol was dependent on specific types of phospholipids surrounding the enzyme. For example, the addition of cholesterol to the bilayers of distearoylphosphatidylcholine decreased enzyme activity but increased stability of the enzyme against thermal denaturation, whereas cholesterol in the bilayers of dioleoylphosphatidylcholine showed little effect on the properties of the enzyme. In order to address the effects of lipid rafts on the activity and stability of UGTs, it is worthwhile to compare the effects of cholesterol in the contexts of raft associated lipids and phospholipids.

Because lipid rafts have been extensively studied with much evidence supporting their existence in living cells, technologies have been developed to monitor raft dynamics such as single molecule tracking together with advanced microscopic analyses [94,97]. These technologies allow researchers to precisely follow the dynamic interactions between raft lipids and raft-associated proteins despite the crowding environment within a cell, and so could be extended to UGTs to follow their interactions with microsomal lipids in real time. A possible experiment would be to use a recombinant system, such as human embryonic kidney 293 (HEK293) cells, where a UGT enzyme and a raft lipid can each be tagged with a Fab-conjugated fluorescent molecule. This would be followed by fluorescence microscopy that records the trajectories of the labeled molecules. The measurement on the time length of co-localization between the enzyme and raft lipid could indicate whether there is a significant interaction between them.

The challenges of using purified membrane protein preparations on the outcome of these experiments has been mentioned several times by Zakim’s group [86,87,90]. Integral membrane proteins tend to associate with detergents and other membrane components after they have been isolated, and form mixed micelles with the detergents and phospholipids used to reconstitute the enzymes. Therefore, the properties displayed by the reconstituted GT_2p_ may be artificially affected by other components more than the phosphatidylcholine variants, and may not faithfully reveal UGT–phospholipid interactions. Also, the native structure and function of membrane proteins might be altered during purification before the enzymes are reconstituted into a new lipid environment. Lastly, if UGTs truly have a significant interaction with surrounding phospholipids in the ER membrane, then it is essential that the enzymes are reconstituted into a native lipid environment for functional studies [87]. The studies reported by Zakim and colleagues on the conformational change of UGTs, especially GT_2p_ reconstituted in phosphatidylcholine variants, were mainly carried out on pig liver microsomes, since the activity of *p*-nitrophenol UGTs was greater than that from cow liver microsomes and so was easier to measure [85]. It would be interesting to carry out these studies using liver from other species, particularly humans, to investigate if lipid–protein interaction is a universal mechanism in the regulation of UGT activity, and how much variation among different species that the influence of the lipid environment could have on the enzymes. Nevertheless, Vessey and Zakim observed latency among all the species examined: guinea pigs, mice, rats, cows, rabbits, and humans, using phospholipase A and the detergent *p*-chloromercuribenzoate as the membrane perturbants [101]. The extent of activation by the addition of either reagent varied among the species. Additionally, most of the kinetic assays were carried out using the substrate *p*-nitrophenol which can be glucuronidated by more than one isoform such as UGT1A6 and 1A1 [102]. This means that the kinetic properties revealed by UGTs could reflect the action of one or multiple isomers. Since protein–protein interactions exist among UGT isoforms, then after altering the lipid phase by changing temperature, the measured kinetic data may not solely represent the regulation from lipid-protein interactions. Above all, the superfamily of UGTs contains many isoforms with overlapping substrate specificity, and these enzymes are widely distributed in the nature from coral and insects to mammals [1]. It is especially important to take these properties into consideration when measuring UGT activity.

It is true that the compartmentation hypothesis is more widely accepted in the field, however the studies by Zakim and colleagues, and others, provide a rich reservoir of data that do indicate that the regulation of UGT activity within the ER membrane environment is complex and may well involve multiple mechanisms.

## 5. Adenine Nucleotide Inhibition Effect

Recently, the inhibitory effects of adenine and adenine-containing nucleotides on UGT activities have been proposed as another explanation of latency [103,104,105]. The first report came from Hallinan et al. who demonstrated that activities of UGTs toward *p-*nitrophenol, estradiol, and estrone were suppressed by 4 mM ATP (adenosine triphosphate) in guinea pig liver microsomes [104,106]. Then, Nishimura et al. found that adenine, ATP, NAD^+^ (nicotinamide adenine dinucleotide, oxidized), and NADP^+^ (nicotinamide adenine dinucleotide phosphate, oxidized) inhibited the glucuronidation of 4-MU and estradiol by allosteric binding to UGTs at a site independent from the substrate and co-substrate binding sites, and proposed that membrane perturbations could lead to the release of these inhibitors and hence an increase in glucuronidation activities [105]. AMP (adenosine monophosphate) by itself barely inhibited UGTs, but decreased the potency of inhibitory nucleotides. It is likely that AMP antagonizes the inhibitory nucleotides by competing for the allosteric binding site, as suggested in a later publication [103]. When Nishimura et al. tested 4-MU UGT activity in rat liver microsomes, latency and inhibitory effects of ATP and NADP^+^ were observed on the UGTs after Brij58 solubilization of the membranes, which suggests the allosteric binding site is luminal [105]. This evidence indirectly supports the compartmentation model since adenine nucleotides must pass the membrane barrier if the UGTs exhibit the native topology, except that the compartmentation model was originally rationalized upon the limited supply of UDPGA for glucuronidation reactions. Lastly, human UGT is also regulated by adenine nucleotides [105]. In microsomes isolated from human embryonic kidney 293 (HEK293) cells that express human UGT1A1, the glucuronidation activity towards estradiol was gradually inhibited by increasing concentrations of ATP, NAD^+^, or NADP^+^. 

In 2012, Ishii et al. compared alamethicin- and Brij58-treated microsomes from rat and human livers, and showed the inhibitory potency of adenine nucleotides was lower in the alamethicin-disrupted microsomes [103]. The luminal concentration of ATP in rat liver ER can be as high as 30 μM which is similar to the IC_50_ value measured from Brij58-treated human liver microsomes (33.8 μM), but much lower than that from rat liver microsomes (66.8 μM). Therefore, under physiological conditions, the level of ATP in the ER lumen might be sufficient to suppress UGT activity. These authors also showed that Brij58 and alamethicin treatments could lead to the release of ATP from rat liver microsomes in vitro. Two structural components have been thought to be important for inhibitory nucleotides to bind the allosteric effector site of UGTs [104,105]. The first one is the adenine skeleton. This is because AMP decreased the inhibitory effects of ATP, adenine, NAD^+^, and NADP^+^, and so was thought to compete with the other adenine nucleotides for the common allosteric binding site. The second structural component is a di- or triphosphate moiety attached to the 5’-position of ribose, because neither AMP nor adenosine showed an inhibitory effect on the UGT activity. However, because adenine was able to inhibit UGT and lacks a phosphate group, the significance of the number of phosphates attached to ribose on the inhibitory effect of adenine nucleotides is called into question. Besides adenine-containing nucleotides, GTP and CTP are the only guanine- and cytidine nucleotides that inhibited UGTs, respectively, using 4-MU as the aglycone substrate [104,105]. CTP and ATP had similar IC_50_ values, and so comparable inhibitory effects. GTP, however, was much less potent than ATP.

UDP is a well-known inhibitor of UGTs. However, unlike adenine nucleotides, UDP is believed to compete with the co-substrate UDPGA for binding to UGTs, hence exerting the inhibitory effect on glucuronidation [104,107,108]. The inhibition of UGT by UDP can be prevented by the inclusion of a divalent metal ion in the incubation mixture, for example 10 mM Mg^2+^.

Despite being a possible explanation of latency, the adenine nucleotides, particularly NADP^+^, could play a role in the regulation of glucocorticoid levels [105,109]. Specifically, 11 β-hydroxysteroid dehydrogenase reduces NADP^+^ to produce cortisone from cortisol in hepatic ER lumen. As the level of NADP^+^ decreases, the inhibitory effect on the UGTs is also decreased, which enables UGTs to glucuronidate excess cortisol. Above all, information on the ER concentrations of adenine nucleotides relative to their cytosolic concentrations and IC_50_ values is needed to further investigate the physiological effects of the nucleotides on glucuronidation and metabolism.

## 6. Conclusions and Future Direction

The controversy over the underlying mechanisms that explain the latency of UGT activity that is observed in vitro in microsomal preparations has continued for more than half a century. It is clear that disruption of the microsomal membrane by physical and chemical treatments is necessary to reveal full UGT enzyme activity in vitro. Based on the available evidence, it is likely that much of the effect is due to the disruption of the membrane allowing increased access of UDPGA (and potentially also aglycone substrates) to the active site of the enzymes. Critical evidence for this comes from the existence of transporter(s) for UDPGA that presumably regulate access of the co-substrate to the enzyme in vivo. However, it is equally clear that UGT enzyme activity can be regulated by membrane components including phospholipids, cholesterol and indeed other proteins, including other UGTs and CYPs. Some of the effects of membrane disrupting agents such as detergents may well be mediated through their impact on the membrane environment as well as on the UGT proteins themselves. 

Readers may ask “why does this matter?”, particularly in a time when we know much about the biology, genetics and regulation of UGTs and glucuronidation. One of the main reasons it remains important is that we are still unable to accurately extrapolate in vitro measurements of UGT activity to estimates of in vivo clearance of drugs that are metabolized by glucuronidation. This in vitro–in vivo extrapolation (IVIVE) consistently underpredicts clearance in vivo for such drugs despite significant advances in physiologically-based pharmacokinetic (PBBK) analysis and a far more sophisticated understanding of the physiological factors that influence UGT activity [110,111]. We believe that increasing our understanding of the underlying mechanisms of UGT latency will help to further refine IVIVE for many drugs that are glucuronidated in humans. Advances in analytical tools such as Förster resonance energy transfer, mass spectrometry, etc. provide the technological basis for advancing our knowledge of this important feature of one of the body’s key chemical defense mechanisms.
